# Effective removal of antibiotics from aqueous solutions by a robust activated carbon: experimental and theoretical study

**DOI:** 10.1039/d5ra05396j

**Published:** 2026-02-16

**Authors:** Khan Badshah, Qaisar Ali, Rashid Ahmad, Iftikhar Ahmad

**Affiliations:** a Department of Chemistry, University of Malakand Chakdara Dir (L) Pakistan; b Center for Computational Materials Science, University of Malakand Pakistan rashmad@gmail.com +923335104105; c Department of Physics, University of Malakand Chakdara Dir (L) Pakistan ahma5532@gmail.com +923155553111

## Abstract

The continuous discharge of antibiotics into water bodies is a potential threat to the ecosystem. We prepared a coal-based activated carbon and characterized it *via* BET, SEM, FTIR spectroscopy, XRD, TEM, TGA and Raman spectroscopy. Its surface area was 3470 m^2^ g^−1^, and its pore size and volume were 1.808 nm and 1.568 cm^3^ g^−1^, respectively. It was evaluated for the removal of moxifloxacin (MOX) and linezolid (LINZ) from water using the Freundlich, Langmuir Temkin and D–R isotherm models and the pseudo-second-order kinetic model. This material removed ∼99.5% of antibiotics, with adsorption capacities of 839 and 832 mg g^−1^ for MOX and LINZ, respectively. Thermodynamic parameters *i.e.* change in Gibb's free energy (Δ*G* = −11.248 to −24.376 kJ mol^−1^), enthalpy (Δ*H* = 185.52 kJ mol^−1^) and entropy (Δ*S* = 263 J mol^−1^ K^−1^) and Δ*G* (−9.52 to −13.818 kJ mol^−1^), Δ*H* (62.36 kJ mol^−1^) and Δ*S* (108 J mol^−1^ K^−1^) reveals spontaneous and endothermic nature of adsorption for MOX and LINZ respectively. The material is very effective in real water samples and is recyclable, demonstrating its stability and reusability. The DFT results, including band gaps, the *E*_HOMO–LUMO_ energy gap, density of states, atoms-in-molecules analysis and charge transfer phenomena, revealed the presence of hydrogen bonding and electrostatic interactions. The study highlights that this material is a potential scavenger of emerging contaminants in water.

## Abstract 2

Activated carbon with an ultra-high surface area 3470 m^2^ g^−1^ is synthesized from coal, showing excellent adsorption capacities for moxifloxacin and linezolid. The DFT study demonstrated that hydrogen bonding is the dominant interaction.

## Graphical abstract

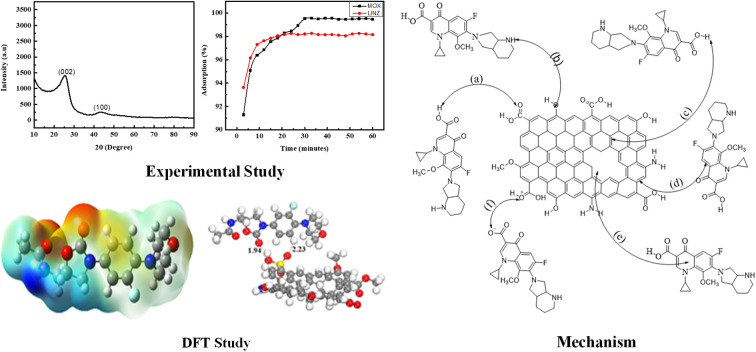

## 1 Introduction

The alarming occurrences of emerging contaminants, such as pharmaceuticals, pesticides and personal care products, in water are a serious threat to the biosphere.^1^ Antibiotics have improved the quality of life, but their discharge into water bodies has increased and their residues in the environment are classified as emerging pollutants.^2^ The main sources of antibiotic discharge into water bodies are municipal and hospital wastes, agriculture and aquaculture and manufacturing units.^3^ This causes antibiotic resistance and other chronic effects including allergic reactions, nephrotoxicity, and neurological and endocrine disorders.^4^ Considering the impacts of antibiotics on the biosphere, there is an urgent need to remove these pollutants from environmental matrices.

Various techniques, *e.g.* bioremediation,^5^ advanced oxidation processes,^6^ and ozonation,^7^ have been reported for the abatement of antibiotics. However, these approaches are associated with limitations such as inefficiency, complicated procedures and high energy demands.^8^ Adsorption is superior due to its simple design, environmental friendliness, great performance and flexible nature.^9^ Various natural and synthetic adsorbents, *e.g.* carbon-based materials, microporous coordination polymers and resins,^10^ clays and minerals, and metal–organic frameworks,^11^ are explored for antibiotic adsorption. However, efficiency, uptake rate and cost limit their large-scale applicability.

An adsorbent with high adsorption capacity, availability, scalability, ecofriendly nature and cost-effectiveness is always required for practical applications. Activated carbon (AC) having outstanding physico-chemical features^12–14^ can be used as potential adsorbents for antibiotics from water.^15^ Several carbon-containing natural precursors, *e.g.*, lignin, fruit pits, wood, nutshells, different coals, and coconut shells,^16,17^ are used to prepare AC. The characteristic of AC depends on the origin and processing conditions of raw materials.^18^ Different activation methods like physical, chemical and biological modifications using different reagents, *e.g.* acids (H_2_SO_4_ and H_3_PO_4_), ozone (O_3_), bases (NaOH and KOH), salts (Na_2_CO_3_ and AlCl_3_) and plasma treatment,^19^ have been used to prepare AC. We prepared an AC from coal with a large pore volume and an extremely high surface area using KOH as an activating agent. To understand the surface behavior, the atomic structures need to be complemented by analysis of the electronic structure that interacts between the chemical species. Therefore, to predict the surface behavior, an atomistic approach is essential, and DFT is the main tool for understanding the properties of molecules and materials on the atomic scale.^20^

This study addresses the water contamination issue caused by the continuous introduction of antibiotics. The development of a cost-effective and sustainable methodology using a safe, efficient and reusable adsorbent is a challenging task; however, the use of activated carbon has the potential to fulfill this goal. In this study, a robust, coal-based activated carbon, with ultra-high surface area as well as tunable pore size and volume, was prepared and assessed for the uptake of broad-spectrum antibiotics, *e.g.* moxifloxacin and linezolid, from water for multiple cycles to assess cost-effectiveness. Adsorption kinetics, isotherms, thermodynamic models and DFT were used to evaluate the adsorption mechanism and performance of activated carbon.

## 2 Experimental section

### 2.1. Chemicals and reagents

**Fig. 1:**
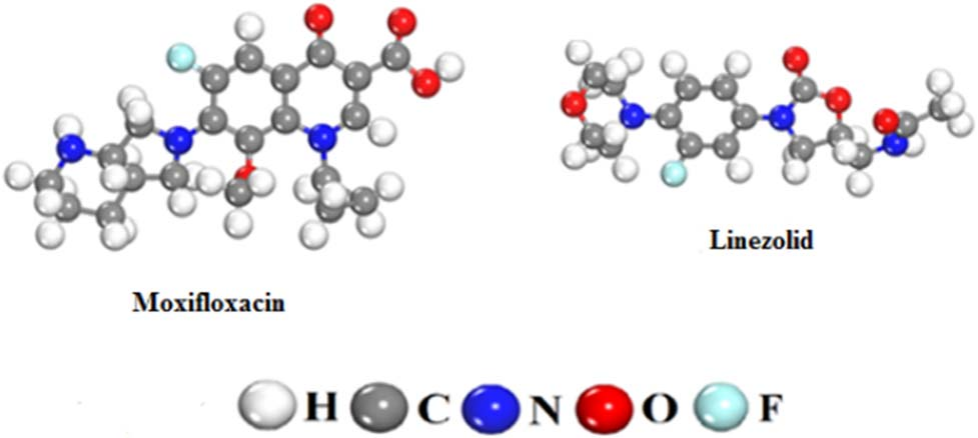
Optimized structures of moxifloxacin and linezolid.

Analytical-grade chemicals, *e.g.* HCl (37%), NaOH (99.9%), KOH (99.9%), CH_3_COOH (99.7%) *etc.*, supplied by Sigma-Aldrich were used in the present study. Antibiotics, moxifloxacin (MF = C_21_H_24_FN_3_O_4_, MW = 401.4 g mol^−1^) and linezolid (MF = C_16_H_20_FN_3_O_4_, MW = 337.35 g mol^−1^) were selected as typical synthetic pollutants. Their optimized structures are presented in Fig. 1.

### 2.2. Preparation and characterization of materials

Coal sample was washed with deionized water and then oven-dried, cooled, crushed (300-mesh) and treated with KOH in a 1 : 4 ratio by weight. The mixture was heated for 2.6 hours at 800 °C in an N_2_ atmosphere in a muffle furnace at the heating rate of 5 °C per minute rise in temperature. The sample was cooled and washed with distilled water followed by a 0.1 M HCl solution to remove the residual KOH. Then, it was dried in an oven for 24 hours at 120 °C and stored under the nomenclature of the coal-based activated carbon (AC). Surface morphology and textural properties, *e.g.* surface area, pore-size distribution and pore volume, were explored *via* TEM, SEM (SU8200 Hitachi SEM Japan) and BET (BET, HORIBA SA-9600 series Japan) at 77 K after degassing the sample in N_2_ for 24 h. The pore-size distribution and specific surface area were calculated using the Brunauer–Emmett–Teller (BET) and Barrett–Joyner–Halenda (BJH) equations. FTIR (PerkinElmer spectrum-one spectrometer; scans, 200; range, 400–4000 cm^−1^; resolution, 4 cm^−1^; detector, liquid nitrogen cooled mercury cadmium telluride), TGA and XRD were used to explore the surface functional groups, thermal stability and crystalline nature of the sample, respectively.

### 2.3. Adsorption procedure

The prepared sample was evaluated for the sequestration of moxifloxacin and linezolid from aqueous solutions. A specific dose of the sample and 10 mL of the MOX and LINZ solutions were added separately to glass culture tubes and shaken on a wrist-action shaker for a given time and then filtered, and the MOX and LINZ residual concentrations in the filtrate were determined using a UV-visible spectrophotometer (Labomed Inc. UVD 2960).^21,22^ The effect of various parameters on the sequestration of MOX and LINZ like sorbent dose (0.002–0.026 g), solute concentration (25–400 mg L^−1^), contact time (3–60 minutes), solution medium (pH, 3–12) and temperature (283–333 K) were optimized in a step-by-step manner. Each result was triplicated under identical experimental conditions, and antibiotic uptake by AC was calculated using the following equations:1
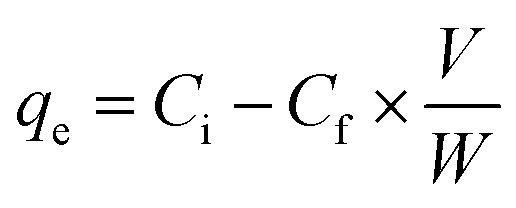
2
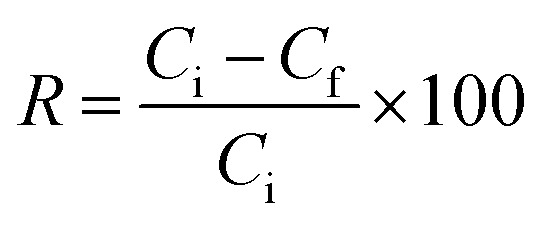
where *q*_e_ (mg g^−1^) denotes the equilibrium adsorption capacity; *C*_i_ (mg L^−1^) and *C*_f_ (mg L^−1^) denote the initial and final solute concentrations; *R* denotes the percent removal; *W* (g) denotes the mass of the adsorbent; and *V* (L) denotes the volume of the solution.

### 2.4. Computational methodology

The principal interactions between antibiotics and activated carbon were examined *via* DFT. The DMOL^3^ simulation package was employed using a hybrid generalized gradient approximation (HGGA) with the Becke, 3-parameter, Lee-Yang-Parr (B3LYP) function in conjunction with the 6-31G basis set.^23,24^ The strength of the interactions/binding energy was computed using the following equation:

3 *E*_ads_ = *E*_adsobate+adsorbent_ − (*E*_adsorbate_ + *E*_adsorbent_)

where *E*_ads, adsorbate+adsorbent_, adsorbate and adsorbent denote the adsorption energy, energies of the complex energy (drug adsorbed on AC), adsorbate (antibiotic) and adsorbent (AC) energies, respectively.

## 3 Results and discussion

### 3.1. Material characterization

#### 3.1.1. XRD analysis

**Fig. 2:**
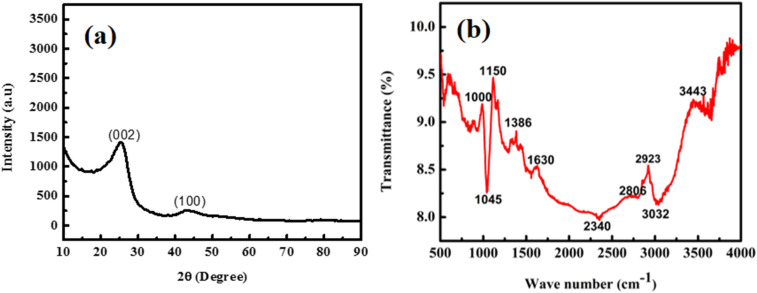
Material characterization XRD (a) and FTIR (b) spectra of activated carbon.

XRD was used to investigate the crystalline nature of the sample (Fig. 2a), the appearance of two peaks at 2*θ* = 25° and 43° corresponding to the (002) and (100) planes, respectively, of graphitic carbon. The broad peaks indicate the highly disordered graphitic nature of activated carbon, illustrating the typical amorphous nature of the prepared sample.^25^ The absence of an extra peak represents the virginity of AC and effective washing of the activating agent (KOH).

#### 3.1.2. FTIR analysis

FTIR was used to explore the surface functional groups, and the various groups present on the carbon skeleton (Fig. 2b) indicated that the peak that appeared at 3443 cm^−1^ was associated with the O–H stretching vibration of the hydroxyl groups. The peak that appeared at 3032, 2923 and 2806 cm^−1^ revealed the C–H stretching vibration of alkene, alkane and aldehyde, respectively. Similarly, the peak that appeared at 2340 and 1630 cm^−1^ was associated with the C≡C and C=C stretching vibrations of alkyne, aromatic alkene and carbonyl groups. The peak that appeared at 1557 and 1386 cm^−1^ represents the N=O and S=O stretching vibration of the nitro and sulfite groups, respectively. The peak observed at 1150, 1045 and 1000 cm^−1^ was associated with the C–O stretching vibration of ester, alcohol, phenol, ether and substituted aromatic ring.^26,27^

#### 3.1.3. BET analysis

**Fig. 3:**
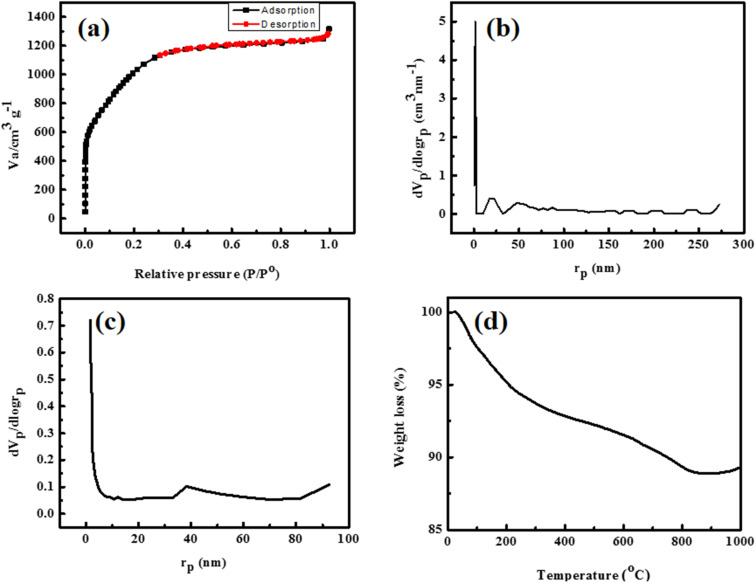
BET adsorption–desorption isotherm (a), BJH plot (b), pore-size distribution (c), and TGA curve (d) of activated carbon.

The nitrogen adsorption–desorption isotherm of activated carbon at 77 K is presented in Fig. 3a and shows that the substrate possesses an ultrahigh surface area (*S*_BET_ = 3470 m^2^ g^−1^) and exhibits the characteristic of type I (b) isotherm. The prompt rise in adsorption volume at a very low relative pressure (*p*/*p*^o^ < 0.01) reflected the micro-porous nature of the surface.^28^ The adsorption curve matched with the type II isotherm at a relative pressure between 0.1 and 0.4, revealing the formation of monolayer followed by multilayer adsorption. At a relative pressure between 0.4 and 1, the adsorption isotherm showed the characteristics of type IV isotherms with H4 hysteresis loops, representing the presence of mesopores.^29,30^ During carbon activation processes, KOH selectively consumed carbon atoms present on the active sites and generated pores. Similarly, when the temperature surpassed the potassium boiling point, the potassium intercalated in the matrix, resulting in an increase in pore volume.^28^ The Barrett–Joyner–Halenda (BJH, Fig. 3b) method was used to calculate the pore-size distributions (PSD). The textural characteristics, *e.g.* total pore volume (*V*_T_ = 1.568 cm^3^ g^−1^), micropore volume (*V*_µ_ = 1.334 cm^3^ g^−1^), mesopore volume (*V*_M_ = 0.234 cm^3^ g^−1^), specific surface area (*S*_µ_ = 1832 m^2^ g^−1^) and external surface area (*S*_ext_ = 1538 m^2^ g^−1^) calculated from the t-plot (Fig. 3C) and BJH plot, are summarized in Table 1. The results suggest that coal is an excellent candidate for preparing an AC with extraordinarily large surface area and tunable pore volume and size.

**Table 1:** Porous structure properties of activated carbon

*S*_BET_ (m^2^ g^−1^)	*S*_µ_ (m^2^ g^−1^)	*S*_ext_ (m^2^ g^−1^)	*V*_T_ (cm^3^ g^−1^)	*V*_µ_ (cm^3^ g^−1^)	*V*_M_ (cm^3^ g^−1^)	*D*_P_ (nm)
3470	1832	1638	1.568	1.334	0.234	1.808

#### 3.1.4. Thermo-gravimetric analysis

The thermal stability and pyrolysis behavior of activated carbon with respect to temperature were explored *via* thermo-gravimetric analysis (TGA), and the results are shown in Fig. 3d. The decomposition of the carbonaceous sample consisted of three stages: dehydration, volatilization and decomposition/degeneration. The first weight loss (5.618%) in the temperature range 20 °C–220 °C occurred due to the evaporation of absorbed moisture. The second weight loss (2.111%) in the temperature range of 221 °C–350 °C is associated with the volatilization of volatile organic compounds, and the third weight loss (2.906%) in the temperature range of 351 °C–800 °C is attributed to the decomposition of oxygen-rich functional groups present in the AC skeleton and degradation of activated carbon (cross-linked fragments).^31,32^ Collectively the total loss (10%) in the temperature range 20 °C–800 °C is low, indicating the highly stable nature of activated carbon.

#### 3.1.5. SEM analysis

**Fig. 4:**
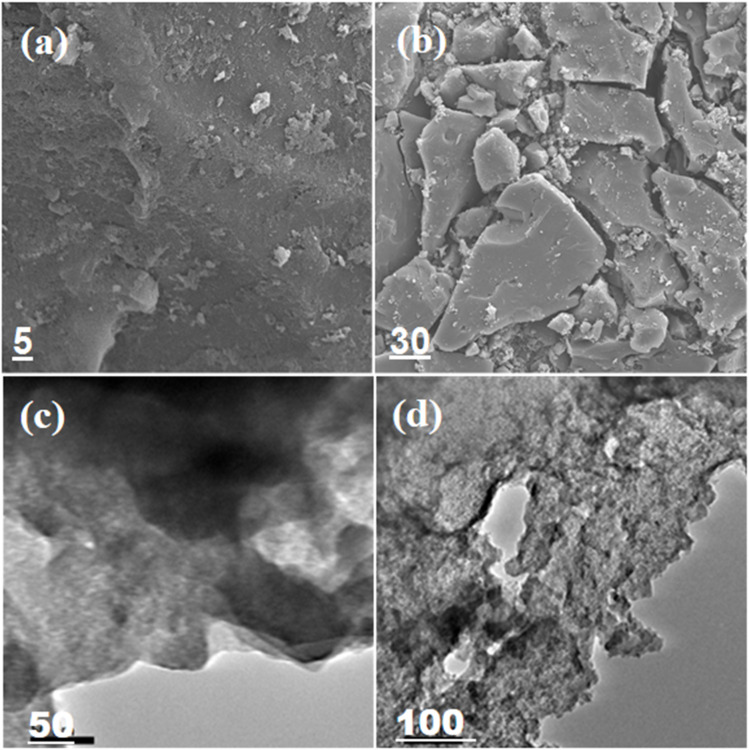
SEM images (a–d) of activated carbon.

The SEM images in Fig. 4a–d show that the sample (AC) possesses a sheet-type morphology containing pores and channels. Furthermore, the images at a high magnification suggest that the texture of activated carbon is quite irregular and full of cavities, supporting the BET and BJH findings.^33^

#### 3.1.6. TEM analysis

**Fig. 5:**
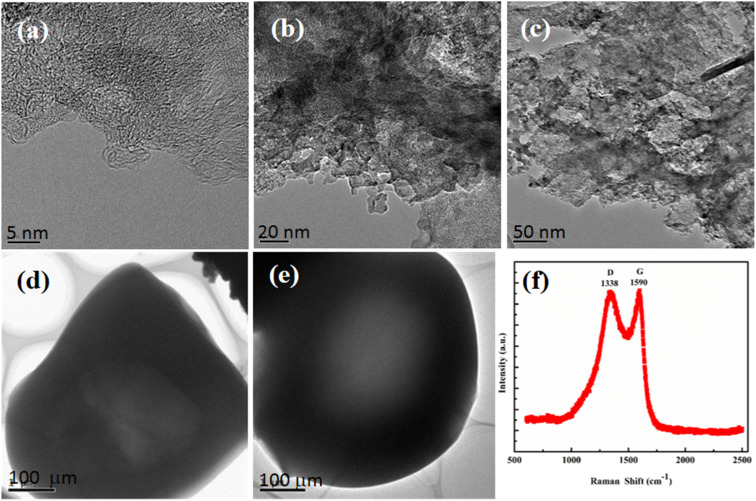
TEM images (a–e) and Raman spectra (f) of activated carbon.

The TEM images of activated carbon are shown in Fig. 5a–e, which describe crumpled and aggregated sheets accompanied by hollow spheres. The image at a high magnification reflects the amorphous and disordered nature of AC. Furthermore, the cross-sectional images revealed channels and pores in the texture of activated carbon, consistent with the BET findings.

#### 3.1.7. Raman spectroscopic analysis

The appearance of two characteristic bands D and G (Fig. 5f) in the Raman spectra of AC confirms the typical amorphous nature. The D-band that appeared at 1338 cm^−1^ reflects the disordered graphitic nature of the material, while the G-band that appeared at 1590 cm^−1^ is consistent of an active Eeg phonon representing sp^2^ bonded carbon atoms respectively. The high intensity ratio of the two bands (D : G) is an indication of more defective sites or high translational symmetry breaking in AC. The prediction based on Raman spectroscopic analysis is consistent with the XRD results.

### 3.2. Antibiotic adsorption study

#### 3.2.1. Effect of the medium on the sequestration of antibiotics

**Fig. 6:**
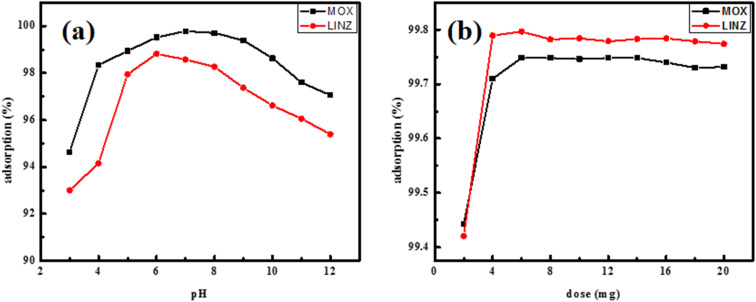
Effect of pH (a) and dosage (b) on moxifloxacin and linezolid sequestration by activated carbon.

The solution medium plays an important role in the adsorptive uptake of antibiotics by influencing the ionization of the adsorbate as well as surface charges.^34^ The adsorption of MOX and LINZ is carried out in a buffer medium having pH 3 to 12, while other parameters, *i.e.* concentration (100 mg L^−1^), dose (0.005 g) and temperature (298 K), were kept fixed; the results are presented in Fig. 6a. The highest uptake of MOX (99.6%) and LINZ (99.315%) occurred at pH 7 and 6, respectively. It has been found that the hydrolysis constants for MOX are (p*K*a_1_ = 6.1 and p*K*a_2_ = 8.7) and exist as positive species (pH < 6.1), negative species (pH > 8.7) and zwitterion (6.1 < pH < 8.7). Solution having pH 7, MOX molecules exist in zwitterions form and H-bonding operates, holding MOX with AC surface. The relatively low adsorption in acidic medium is due to the abundance of small H^+^ ions, competing the adsorption of MOX. In basic medium, de-protonation occurs, which results in the repulsive interaction between negatively charged MOX and AC, causing a decrease in MOX uptake.^35^ Similarly, the high uptake of LINZ at pH 6 is due to the hydrogen bonding interaction between LINZ and the AC surface.

#### 3.2.2. Effect of sorbent dose on sequestration

The adsorption rate is affected by the massive interaction between the adsorbate and adsorbent. Therefore, it was tested in the range of 0.002 to 0.026 g at an optimized pH. The results (Fig. 6b) show that the adsorption rate increases for MOX (up to 99.76%) and LINZ (up to 99.75%), with increases in sorbent dose from 0.002 g to 0.006 g (0.2 g L^−1^ to 0.60 g L^−1^) and then remaining unchanged up to 0.02 g. The increase in the removal rate with the dose is due to the increase in the accessibility of the active sites.^36,37^

#### 3.2.3. Effect of contact time on antibiotic removal

**Fig. 7:**
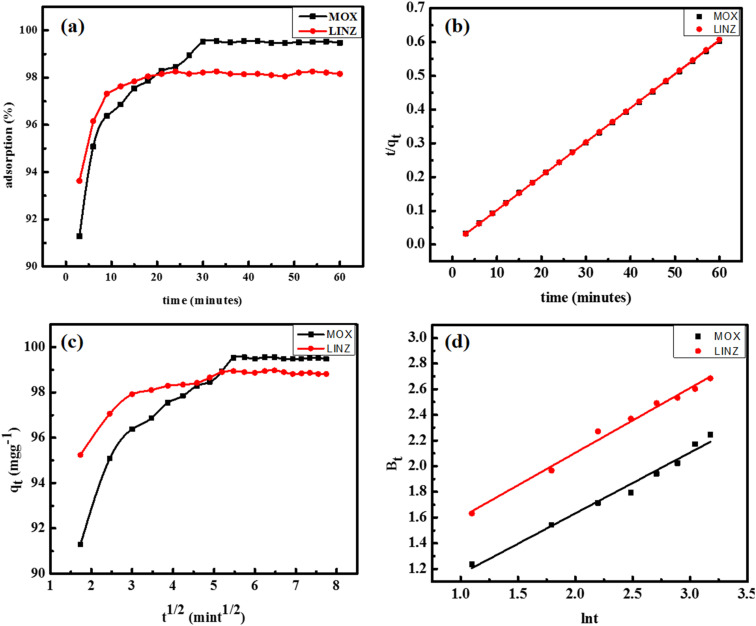
Effect of shaking time (a) and the pseudo-second-order (b), Morris–Webber (c), and liquid film diffusion (d) kinetic plots of moxifloxacin and linezolid adsorbed onto activated carbon.

It is an important parameter that plays a vital role in the commercial application of the material. The time effect was studied in the range of 3 to 60 minutes (Fig. 7a). The equilibrium was established within 30 and 25 minutes for MOX (99.6%) and LINZ (99.5%), respectively. The very fast adsorption rate (>98%) occurs within 9 minutes, representing the high affinity of the adsorbent for the adsorbate. The fast uptake rate is due to the availability of bare active sites at initial stage and on occupation of active sites the rate of adsorption slowdown due to the hindrance offered by occupied sites.^38,39^

Adsorption involves the mass transfer of solute to the solid surface. To understand the mechanism, pseudo-first- and second-order kinetic models^38,40^ were applied:

5 ln(*q*_e_ − *q*_*t*_) = ln *q*_e_ + *k*_1_*t*

6
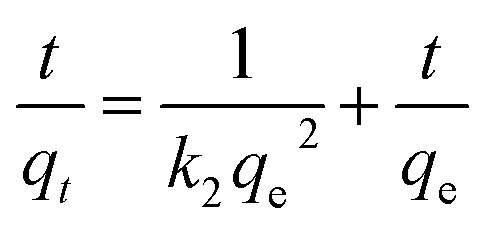
where *q*_e_ (mg g^−1^) and *q*_*t*_ (mg g^−1^) denote the equilibrium adsorption and adsorption at time *t* (minutes), respectively. *k*_1_ (mint^−1^) and *k*_2_ (g mg^−1^ mint^−1^) are the rate constants of pseudo-first- and pseudo-second-order, respectively. Plotting ln (*q*_e_–*q*_*t*_) *vs. t* gives a pseudo-first-order plot (Fig. S1) and *t*/*q*_*t*_*vs. t* gives a pseudo-second-order plot (Fig. 7b). In Table 2, the kinetic parameters computed from the slope and intercept values are given. The pseudo-second-order kinetic model is best fitted to experimental data with regression coefficient (*R*^2^ = 0.99) and rate constant (*k*_2_ = 0.028 and 0.121 g mg^−1^ mint^−1^) for MOX and LINZ, respectively. The experimental adsorption capacity values (*q*_e, exp_ = 99.594 and 99.321 mg g^−1^) and calculated adsorption capacity (*q*_e,calc._ = 100.2 and 100.01 mg g^−1^) for MOX and LINZ respectively are very close to each other indicating the adsorption of MOX and LINZ onto AC follow pseudo 2nd order kinetic model.

**Table 2:** Kinetic parameters of moxifloxacin and linezolid adsorbed onto activated carbon

Kinetic model	Parameters	Moxifloxacin	Linezolid
Pseudo second order	*k*_2_ (g mg^−1^ min^−1^)	0.028	0.121
	*q*_e_ (experimental, mg g^−1^)	99.594	99.321
	*q*_e (_calculated, mg g^−1^)	100.20	100.01
	*R* ^2^	0.999	0.999
Pseudo first order	*k*_1_ (min^−1^)	−0.091	−0.039
	*q*_e_ (mg g^−1^)	65.709	23.416
	*R* ^2^	0.588	0.764
Morris–Webber	*k*_id_ (mg g^−1^ min^−1^)	1.02	0.398
	*C*	92.81	96.3
	*R* ^2^	0.738	0.608
Richenberg's model	*k*_R_ (min^−1^)	0.273	0.177
	*A*	0.744	0.177
	*R* ^2^	0.979	0.990

Besides, the movement of the adsorbate from the bulk phase to the film surrounding the adsorbent, the adsorption process is described in three stages:^39^ (a) external mass transfer of solute to the exterior surface of adsorbent across the liquid film, called film diffusion (outer diffusion/boundary layer diffusion); (b) transport of adsorbate from the outer surface to the pores/capillaries of the adsorbent internal structure, called intraparticle or inner diffusion; and (c) adsorption on the active sites in the inner or outer surface of adsorbent. The third step is very fast and is not considered as a rate-limiting or rate-controlling step. Commonly the adsorption rate is controlled by inner or outer diffusion or both that occurs simultaneously. The rate-controlling step was determined by subjecting experimental data to the Morris–Webber and Richenberg's (Boyd) models^41^ given below:7
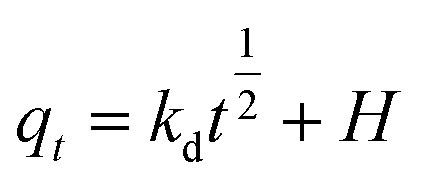
8
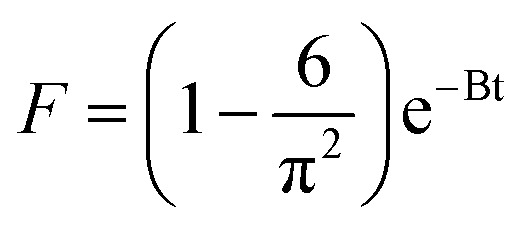
where *k*_d_ (mg g^−1^ mint^−1^) is the rate constant of intraparticle diffusion and *H* (mg g^−1^ mint^−1/2^) is the thickness of the boundary layer. *F* is the ratio of *q*_*t*_ to *q*_e_ (*q*_*t*_/*q*_e_), and Bt is a mathematical function. The Morris–Webber plot (*q*_*t*_*vs. t*^1/2^, Fig. 7c) shows that the line does not pass through the origin reveals that intra particle diffusion is not the sole rate limiting step. Richenberg's model is very useful in differentiating the external mass transfer and intraparticle diffusion mechanism. The plot of Bt *vs. t* is linear (Fig. 7d); however, it does not pass through the origin, indicating that initially, the rate of moxifloxacin and linezolid adsorption onto activated carbon is controlled by intraparticle diffusion followed by film diffusion.

#### 3.2.4. Effect of antibiotic concentration on their sequestration

**Fig. 8:**
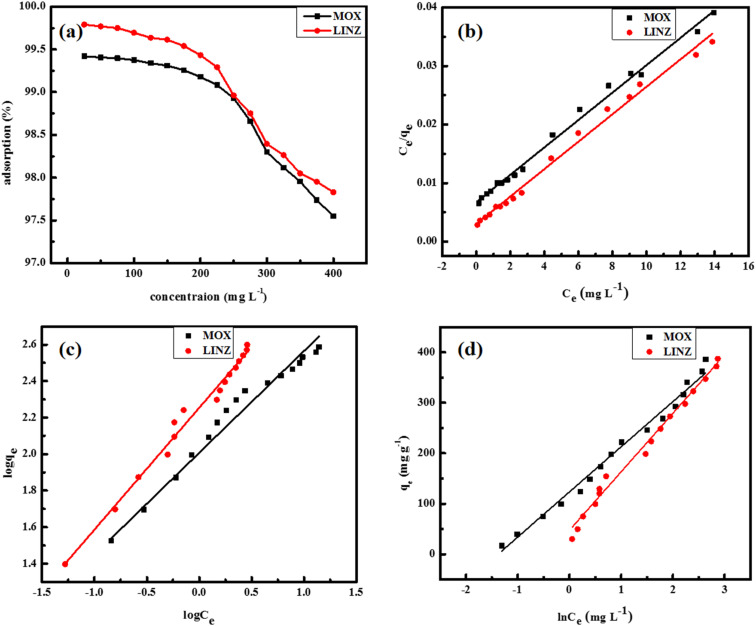
Effect of antibiotics concentration (a) and the adsorption isotherms, Langmuir (b), Freundlich (c), and Temkin. (d) Plots of moxifloxacin and linezolid adsorbed onto activated carbon.

The effect of solute (antibiotics) concentration on its removal by activated carbon was evaluated in the range of 25–400 mg L^−1^ under optimized conditions (Fig. 8a). The removal rate of the adsorbent was quite high initially and then decreased with the concentration. The increase in the initial concentration of adsorbate molecules increases their ratio to the active sites, causes a decreases in % adsorption.^42^ The increase in the initial concentration provides a driving force to overpower resistance to mass transfer between the phases.^43^

The adsorption mechanism of MOX and LINZ onto AC was explored by subjecting the experimental data to adsorption models, *viz.* Freundlich, Langmuir, Dubinin–Radushkevich (D–R) and Temkin models,^44^ which are expressed as follows:9
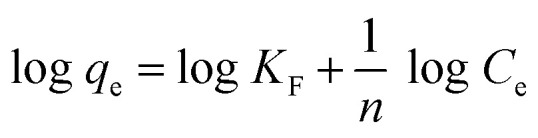
10
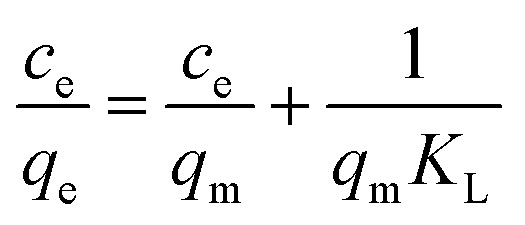

11 ln *q*_e_ = ln *x*_m_ − *βε*^2^

12 *q*_e_ = *β*_T_ ln *K*_T_ + *β*_T_ ln *C*_e_

The *R*_LG_, *E*_DR_ and *b*_T_ parameters were calculated using the following equations:13
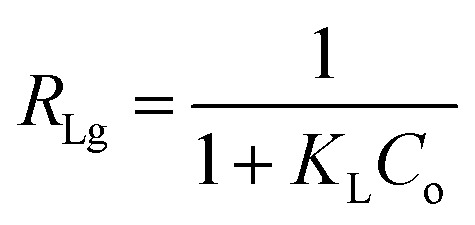
14
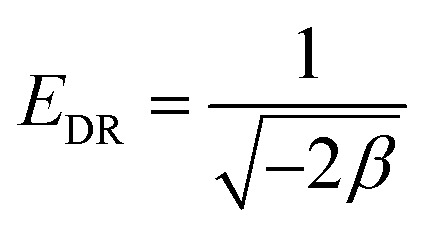
15
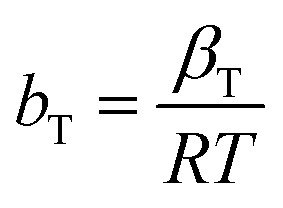
where *R*_LG_, *E*_DR_ and *b*_T_ are the Langmuir, D–R and Temkin parameters, respectively. *C*_o_ (mg L^−1^) and *C*_e_ (mg L^−1^) are the initial and equilibrium concentrations. *K*_F_ (mg g^−1^ mg^−1/*n*^ L^1/*n*^), *K*_L_ (L mg^−1^) and *K*_T_ (L mg^−1^) are the Freundlich, Langmuir and Temkin constants representing the adsorption capacity, affinity of adsorbate towards the adsorbent and binding energy constant, respectively. *q*_m_ (mg g^−1^) and *x*_m_ (mg g^−1^) are Langmuir and D–R parameters that describe the maximum adsorption capacity and theoretical adsorption capacity, respectively. 1/*n* is the Freundlich constant representing the adsorption intensity, and *β*_T_ (J mol^−1^) is the Temkin parameter representing the heat of sorption. *β* (kJ^2^ mol^−2^) represents the constant related to the sorption free energy of the sorbate as it migrates to the sorbent surface from infinite distance, and *ε* is a Polanyi potential. *T* is the temperature (*K*) and *R* (8.314 J mol^−1^ K^−1^) is the universal gas constant.

Langmuir isotherm considers a homogeneous surface with energetically equal binding sites and no transmigration of adsorbed molecules. The Freundlich model assumes heterogeneous surface associated with energetically non-equivalent active sites. The Temkin model accounts for the strength of the interaction between the adsorbate, adsorbent and adsorption energy. The Dubinin Radushkevich model is used to distinguish physisorption and chemisorption. The Freundlich, Langmuir, Dubinin Radushkevich and Temkin isotherm parameters calculated from the slope and intercept (Fig. 8b–d and S2) are given in Table 3. The Langmuir model yielded regression coefficients (*R*^2^) of 0.985 and 0.984 and adsorption capacities (*q*_m_) of 839 and 832 mg g^−1^ for MOX and LINZ, respectively, indicating homogeneous nature and monolayer adsorption. The adsorption capacity (*q*_m_) of AC for MOX and LINZ is far higher than that of the reported adsorbent (Table 4). The Freundlich constant (1/*n* < 1) indicates the favourable nature of MOX and LINZ adsorption onto AC, and the Temkin parameter (*b*_T_ = 27.5 and 21.4 J mol^−1^) indicates the high affinity of adsorbent for adsorbate. The intermediate value of heat of sorption (*β*_T_ = 89.93 and 115.78 kJ mol^−1^) for MOX and LINZ reveals the hybrid nature of adsorption.

**Table 3:** Adsorption isotherm parameters of moxifloxacin and linezolid adsorbed onto activated carbon

Isotherms	Parameters	Moxifloxacin	Linezolid
Langmuir	*q*_m_ (mg g^−1^)	839	832
	*K*_L_ (10^−5^ L mg^−1^)	0.34	0.828
	*R*_Lg_	0.014	0.006
	*R* ^2^	0.985	0.984
D–R	*X*_m_ (mg g^−1^)	275	314
	*β* (10^−4^) (kJ^2^ mol^−2^)	0.025	0.133
	*E*_DR_ (kJ mol^−1^)	4.472	2.103
	*R* ^2^	0.831	0.834
Freundlich	*K*_F_ (mg g^−1^ mg^−1/*n*^ L^1/*n*^)	7.440	9.516
	1/*n*	0.557	0.666
	*R* ^2^	0.959	0.984
Temkin	*K*_T_ (L mg^−1^)	3.954	1.509
	*b*_T_ (J mol^−1^)	27.553	21.401
	*β*_T_ (kJ mol^−1^)	89.93	115.78
	*R* ^2^	0.984	0.972

**Table 4:** Moxifloxacin and linezolid adsorption capacities of different adsorbents

Adsorbent	Adsorbate	I. C (mg L^−1^)	*q*_max_ (mg g^−1^)	References
Biochar	MOX	40–140	39.10	45
MOF-808-SIPA	MOX	0.1–2.0	287.1	46
MNPs	MOX	10–60	39.06	47
RmGO/PANI	MOX	150–525	27.33	48
MSPNPs	MOX	5–25	28.57	49
MPNPs	MOX	5–25	24.32	49
MgONPs	LINZ	10–100	123.45	50
ZnO–MgONCs	LINZ	10–100	140.28	50
AC	MOX	25–400	839	This work
AC	LINZ	25–400	832	This work

#### 3.2.5. Effect of temperature on antibiotic sequestration

**Fig. 9:**
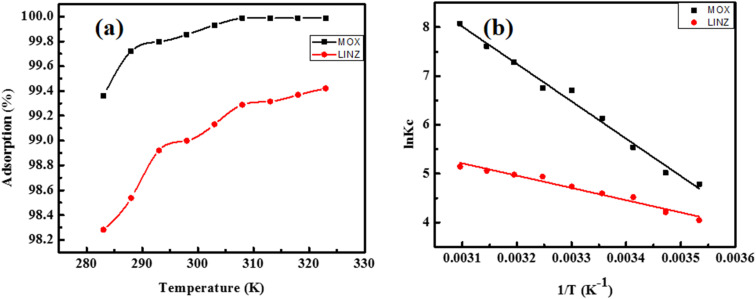
Effect of temperature (a) and vant Hoff plot (b) of moxifloxacin and linezolid adsorbed onto activated carbon.

Temperature is an important parameter influencing the adsorption process. Therefore, the removal of MOX and LINZ by AC was studied at different temperatures (283 to 323 K), and the results are given in Fig. 9a. The % adsorption of MOX and LINZ increased with the temperature, reflecting its endothermic nature. The high uptake rate with rise in temperature is accelerated by the movement of solute molecules from liquid bulk phase to surface followed by diffusion into active sites and the necessary activation energy to overcome the energy barrier.^51–53^ Adsorption is generally an exothermic process; however considering the solvent effect, water molecules initially adsorb onto the surface, and for antibiotic adsorption, they must be desorbed. The desorption of water molecules is an endothermic reaction, and the heat absorbed during water desorption exceeds the heat released during antibiotic adsorption. It has been reported that about 28.6 to 50.78 kJ mol^−1^ of energy is required for water desorption and is affected by surface functionality and textural properties like pore volume *etc.*^54,55^ Additionally, the molar volume of water molecules is much smaller than that of antibiotic, meaning numerous water molecules must be displaced to accommodate a single antibiotic molecule. Similar thermodynamic results have been reported by other researchers.^56,57^ The thermodynamic parameters, *i.e.* change in enthalpy (Δ*H*, kJ mol^−1^), Gibbs free energy (Δ*G*, kJ mol^−1^) and entropy (Δ*S*, J mol^−1^ K^−1^), were calculated using the following equations:^58^

16 Δ*G* = −*RT* ln *K*_c_

17
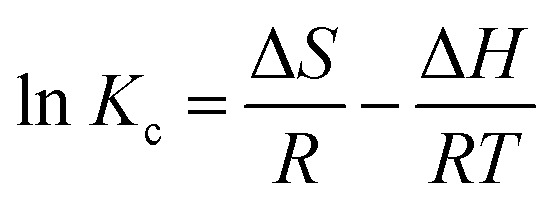
where *R* (8.314 J mol^−1^ K^−1^), *T* (*K*) and *K*_c_ are universal gas constant, absolute temperature and the adsorption partition coefficient, respectively. The thermodynamic parameters (Δ*H* and Δ*S*) calculated from the slope and intercept of the vant Hoff plot (Fig. 9b) are given in Table S1. The negative values of the Gibb free energy (Δ*G* < 0) for MOX and LINZ reveal the spontaneous nature of adsorption. The positive value of enthalpy change (Δ*H* = 185 and 62 kJ mol^−1^ for MOX and LINZ, respectively) indicates the endothermic nature of adsorption. The change in entropy (Δ*S* = 263 and 108 J mol^−1^ K^−1^) for MOX and LINZ respectively shows that randomness increases when solute molecules adsorbed on the surface of adsorbent. The thermodynamic parameters indicate that adsorption of MOX and LINZ onto AC is feasible, spontaneous and endothermic in nature.

#### 3.2.6. Effect of real water samples on antibiotic sequestration

The effect of tap water on the adsorption efficiency of adsorbent was explored. Tap water contains a number of dissolved impurities like metals ions, chlorides, sulfates, nitrates, bicarbonates *etc.*, which are responsible for altering water viscosity, density and mass transfer. These contaminants may either remain non-interactive or compete with antibiotics for active sites and cause decreases in adsorption rate. MOX and LINZ were adsorbed onto AC in tap water (Fig. S3), and there was a negligible effect on its adsorption, suggesting the selective nature of AC for MOX and LINZ. This performance suggests the applicability of AC for antibiotics under a wide range of aquatic conditions.

#### 3.2.7. Regeneration of materials

**Fig. 10:**
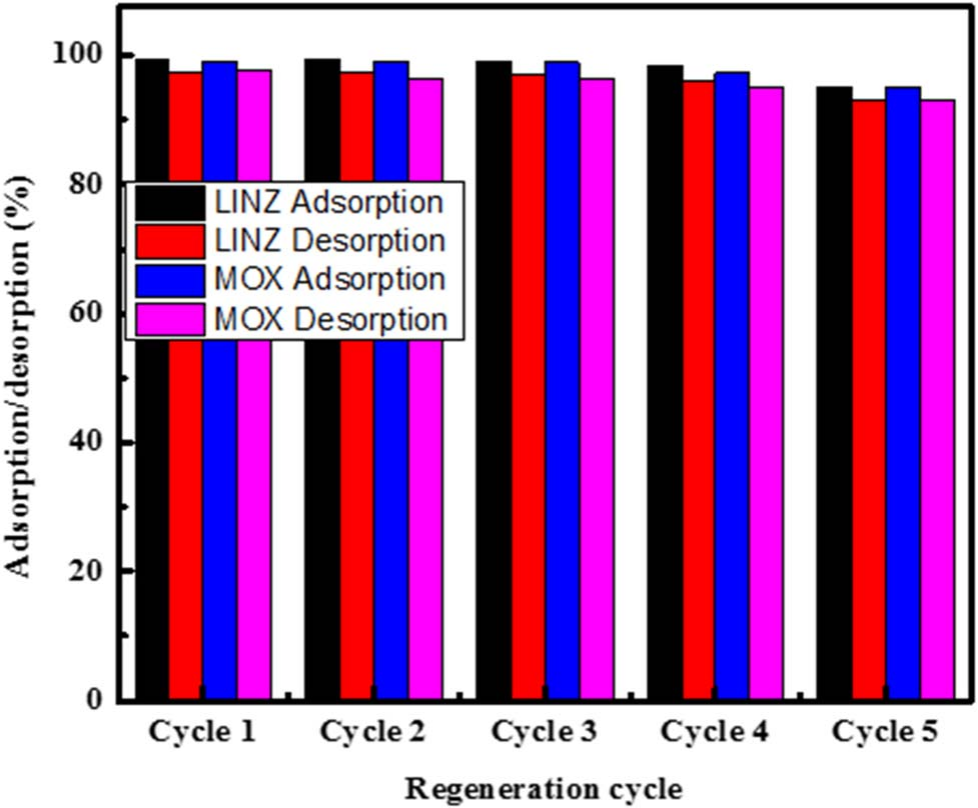
Regeneration cycles of activated carbon.

Adsorbent reusability is an important parameter affecting performance, cost, time, energy and environmental protection. The recyclability of adsorbent associated with such qualities significantly reduces the issues of the synthesis of a new adsorbent and disposal of the old adsorbent. The AC was loaded under previously optimized conditions and then treated with CH_3_CH_2_OH for desorption, and the concentrations of the antibiotics were noted. The desorbed AC was thoroughly rinsed with distilled H_2_O and dried. The same procedure was repeated up to five cycles for the adsorption–desorption of MOX and LINZ, and the results are given in Fig. 10. The results indicate that the adsorption rate of AC dropped from 99.65% to 96% after five successive cycles, demonstrating that AC can be used effectively up to many cycles with no considerable loss in adsorption efficiency.

### 3.3. Adsorption mechanism

Physical and chemical processes are involved in adsorption. A number of factors like the properties of adsorbent/adsorbate and the environment influence the adsorption mechanism. The different surface functional groups of the adsorbent serve as active sites for interactions with antibiotics. Similarly, the ionization of antibiotics are greatly affected by the solution medium. Thus, a number of mechanisms like hydrogen bonding, electrostatic interaction, Yoshida hydrogen bonding, electron–donor–acceptor interaction and pore-filling are involved in adsorption.

**Fig. 11:**
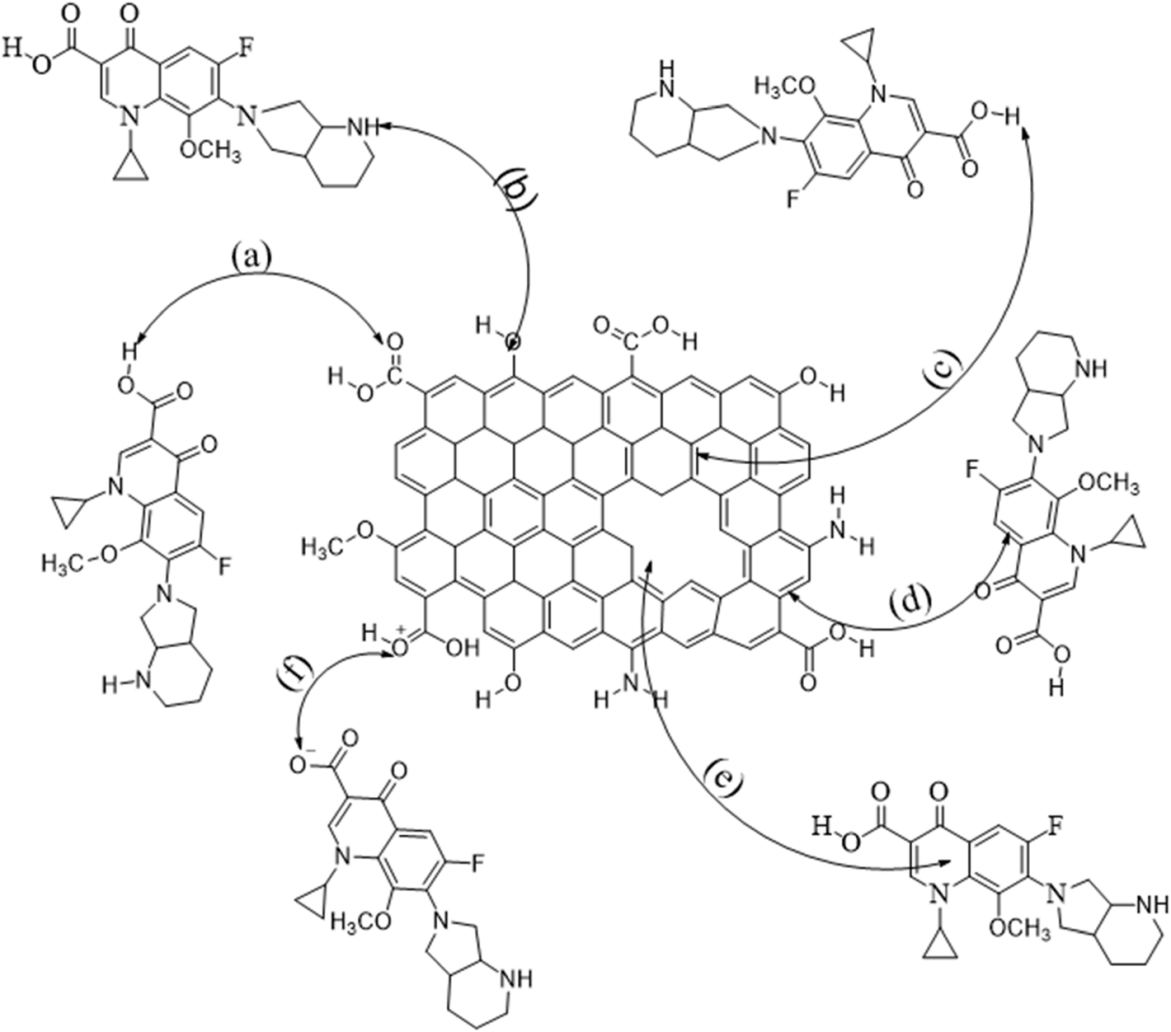
Adsorption mechanisms, hydrogen bonding (a, and b), Yoshida hydrogen bonding (c), electron–donor–acceptor interaction (d), pore filling (e), and electrostatic interaction (f) of antibiotics adsorbed onto activated carbon.

Electrostatic interactions, either attractive or repulsive, are operative during adsorption^59^ and greatly affect the dissociation constant of antibiotics (pKa), solution medium (pH) and point of zero charge of adsorbent (pHpzc). The experimental results (Fig. 4a) show that maximum adsorption occurs at pH 7. The adsorbent is positively charged at pH 7 (pH < pHpzc = 8), whereas the antibiotics is negatively charged (pH > pKa = 6.16); therefore, an electrostatic and attractive adsorption mechanism^60^ is established (Fig. 11). The results (Fig. 6a) shows that pH affects the adsorption processes upto 6.5% reflects that other mechanisms play vital role in the adsorption mechanism.

FTIR analysis revealed that there are nitrogen- and oxygen-containing functional groups like –NH_2_, –C=O, –O–H, –COOH, and –O–CH_3_ on the surface of the adsorbent, whereas antibiotics have –NH, –C=O, –COO, and F functional groups. The presence of these sites on the surface makes H-bonding easier with corresponding antibiotics. Besides, normal hydrogen bonding, such as Yoshida hydrogen bonding,^61^ enables binding of antibiotics with the adsorbent, contributing to the adsorption mechanism.

Antibiotic adsorption onto carbon-based materials are greatly affected by the electron–donor–acceptor phenomenon. Electron–donor–acceptor interactions operate through the electron-rich π system of one species with anions, metals, molecule or other π systems. Antibiotic molecules contain a strong electron-withdrawing F atom and aromatic ring. Activated carbon is rich in π systems. Therefore, antibiotics act as an electron acceptor, while activated carbon act as a donor, enhancing adsorption and contributing to the adsorption mechanism (Fig. 11).

Surface characteristics like surface area as well as pore volume, size and morphology have great impact on adsorption. In physical adsorption, van der Waals forces and pore-filling influence the adsorption mechanism. The tailored pore size of adsorbents enables antibiotics to pass through channels and diffuse into the pores. The decrease in pore volume (up to 0.521 cm^3^ g^−1^) (Fig. S9) after adsorption indicates that the pore-filling mechanism boosts adsorption. DFT studies also confirmed the involvement of H-bonding, electrostatic interaction and weak van der Waal forces in adsorption.

## 4 Conclusions

An activated carbon with a robust surface area (3470 m^2^ g^−1^) was prepared using coal as a precursor and KOH as an activating agent. Porous AC with ultra-high specific surface area, tunable pore size and volume and highly disordered graphitic nature was obtained. AC was used for the abatement of MOX and LINZ from aqueous solutions, and the results indicated that short equilibration time and high adsorption capacity at a wide pH range make AC quite worthy for practical utility. The thermodynamic study suggested the spontaneous and endothermic nature of adsorption. Kinetic models indicated that the rate of adsorption is controlled by intraparticle diffusion and external mass transfer. The regeneration study of the material highlighted the reusability and stability of adsorbent without losing its substantial activity. Antibiotic adsorption in real water indicates the selective nature of adsorbents. The DFT results indicate the hydrogen bonding and electrostatic interactions of MOX and LINZ adsorbed onto AC. The combined experimental and DFT findings suggest that AC is an effective and promising adsorbent for the removal of antibiotics from water.

## Author contributions

Khan Badshah: conceptualization, data curation, formal analysis, methodology, writing first draft. Qaisar Ali: visualization, software, validation. Rashid Ahmad and Iftikhar Ahmad were responsible for resources, investigation, conceptualization, review, project supervision and administration.

## Conflicts of interest

There are no conflicts of interest to declare.

## Abbreviations

### Symbol

*S*_BET_
: Total surface area
*S*_µ_
: Specific surface area
*V*_T_
: Total pore volume
*V*_µ_
: Micropore volume
*V*_M_
: Mesopore volume
*D*_P_
: Average pore diameter
*q*_*t*_
: Adsorption capacity at time t
DFT: Density functional theory
*C*_e_
: Equilibrium concentration
*K*_T_
: Temkin constant
*q*_e_
: Equilibrium adsorption capacity
*q*_max_
: Maximum adsorption capacity
Δ*H*: Enthalpy change
*C*_o_
: Initial concentration
*K*_F_
: Freundlich constant
Δ*G*: Gibbs free energy change
Δ*S*: Entropy change
*x*_m_
: D–R theoretical adsorption capacity
1/*n*: Freundlich constant representing the adsorption intensity
*β*_T_
: Temkin parameter representing the heat of sorption
*β*
: Constant related to free energy of sorption
*R*
: Universal gas constant
*k*_1_
: Pseudo-first-order rate constant
*k*_2_
: Pseudo-second-order rate constant
*q*_e,exp_
: Experimental adsorption equilibrium
*q*_e, calc_
: Calculated adsorption equilibrium
*R*_LG_
: Langmuir parameter
S. A: Surface area
*E*_DR_
: D–R parameter
*ε*
: Polanyi potential
*b*_T_
: Temkin parameter
*R* ^2^
: Regression coefficient
I. C: Initial concentration
*K*_L_
: Langmuir constant
*R*_LG_
: Langmuir parameter
LINZ: Linezolid
*T*
: Temperature
*t*
: Time
AC: Activated carbon
MOX: Moxifloxacin

## Supplementary Material

RA-016-D5RA05396J-s001

## Data Availability

The authors confirm that the data supporting the findings of this study are available within the article and its supplementroy information (SI). Supplementary information: tables, figures and DFT analyses. See DOI: https://doi.org/10.1039/d5ra05396j.
