# Supplementary material for: Effective removal of antibiotics from aqueous solutions by a robust activated carbon: experimental and theoretical study
Source: RSC Adv. 2026 Feb 16;16(10):8875–88. doi: 10.1039/d5ra05396j (PMC12907798; doi:10.1039/d5ra05396j)
Supplement: RA-016-D5RA05396J-s001 [file RA-016-D5RA05396J-s001.pdf]

Supplementary Information (SI) for RSC Advances.
This journal is © The Royal Society of Chemistry 2026

Supplementary Materials

Effective Removal of Antibiotics from Aqueous Solutions by a Robust Activated Carbon: Experimental and Theoretical Study

Khan Badshah [1, 2], Qaisar Ali [2], Rashid Ahmad [*1, 2, 3], Iftikhar Ahmad[* 2, 4]

[1]*Department of Chemistry, University of Malakand, Chakdara Dir (L), Pakistan*

[2]*Center for Computational Materials Science, University of Malakand, Pakistan*

[3]*Vice-Chancellor University of Malakand, Chakdara Dir (L), Pakistan*

[4]*Department of Physics, University of Malakand, Chakdara Dir (L), Pakistan*

*Corresponding Authors: E-mail:rashmad@gmail.com Phone: +923335104105

Email: ahma5532@gmail.com Phone: +923155553111

Table S1 Thermodynamic Parameters of Moxifloxacin and Linezolid Adsorbed onto Adsorbent Activated carbon

T (K)	ΔG ($KJ\ mol^{-1}$)	ΔH ($KJ\ mol^{-1}$)	ΔS ($J\ mol^{-1}\ K^{-1}$)	ΔG ($KJ\ mol^{-1}$)	ΔH ($KJ\ mol^{-1}$)	ΔS ($J\ mol^{-1}\ K^{-1}$)
	moxifloxacin				linezolid	
283.15	-11.248			-9.52		
288.15	-12.008			-10.08		
293.15	-13.483			-11.006		
298.15	-16.190	185.52	263	-11.384	62.36	108
303.15	-16.895			-11.934		
308.15	-17.297			-12.649		
313.15	-18.952			-12.953		
318.15	-20.116			-13.373		
323.15	-24.376			-13.818		

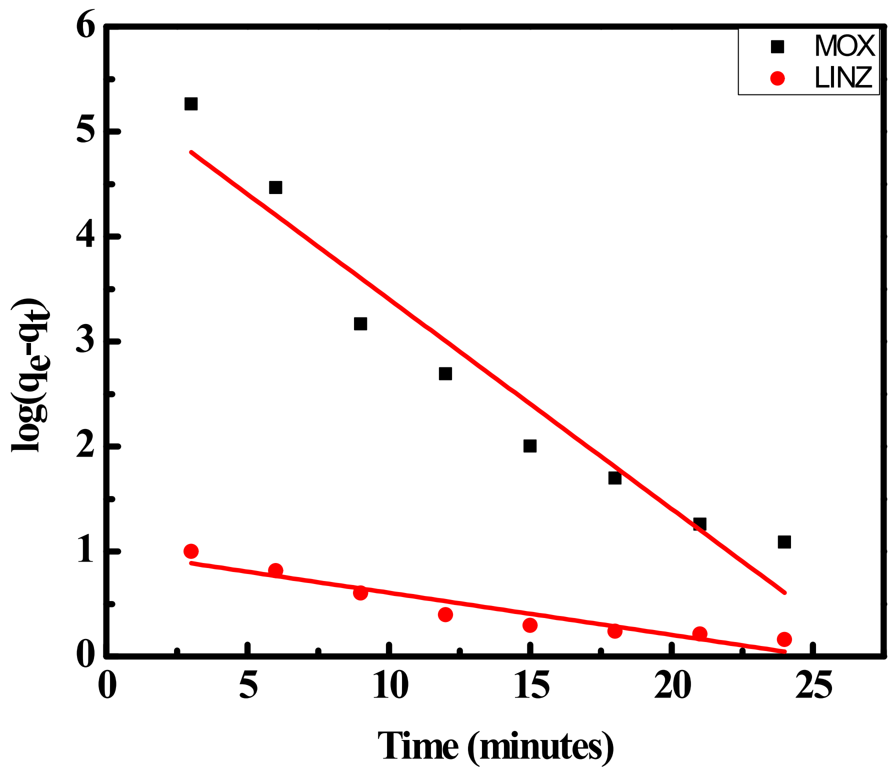

Figure S1 Pseudo 1st Order Kinetic Plot of Moxifloxacin and Linezolid Adsorbed onto Activated Carbon

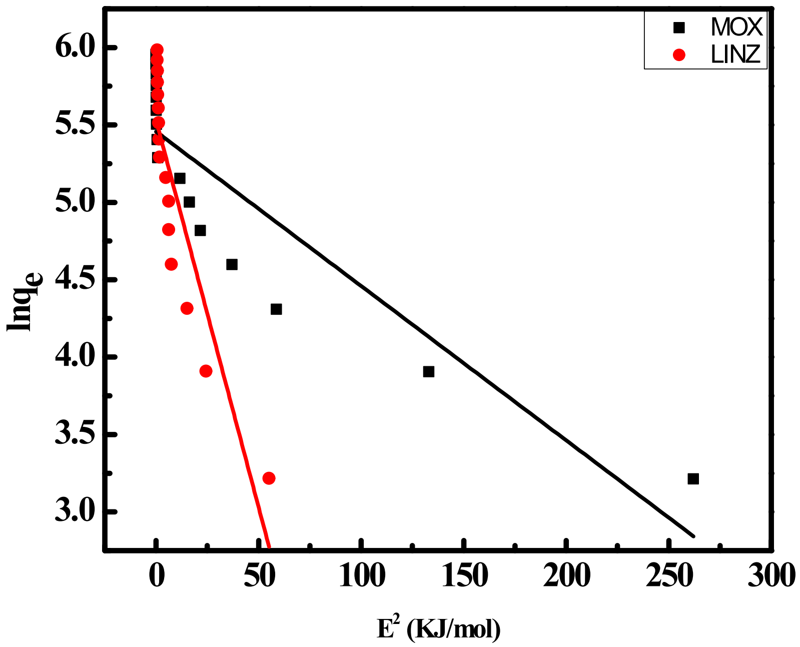

Figure S2 D-R Isotherm Plot of Moxifloxacin and Linezolid Adsorbed onto Activated Carbon

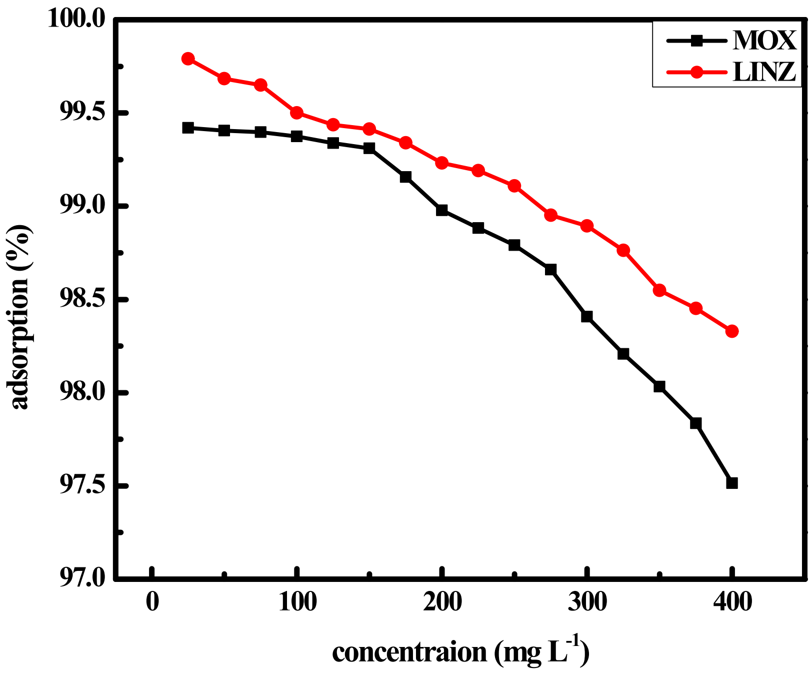

Figure S3 Moxifloxacin and Linezolid Uptake in Tap Water by Activated Carbon

DFT Analysis of Antibiotics Adsorption

For the verification of experimental results and exploration of the step-by-step adsorption mechanism at atomic level, quantum mechanical DFT calculations are crucial.[1] The geometries of activated carbon, linezolid and moxifloxacin were designed and DFT tool is used to compute the interaction involved in the adsorption of MOX and LINZ onto AC using Avogadro software and optimized using $DMOL^3$ suite of program [2, 3] with B3LYP theory employing 6-31G (d, p) basis set and the optimized geometries are shown in Fig. 1 and S4. The nucleophilic and electrophilic centers in the adsorbent and adsorbate were examined using molecular electrostatic potential map (MEP, Fig. S5). The yellow and red color depict electrostatic while blue and green color depict nucleophilic center in the MEP map. The oxygen and fluorine atoms in the AC, linezolid and moxifloxacin are surrounded by red color indicate electrophilic zone whereas the hydrogen atoms

attached to oxygen, Sulphur and carbon atoms demonstrate nucleophilic center surrounded by blue color in the MEP map. The interaction between the electrophilic and nucleophilic centers of the adsorbent and adsorbate were studied. Three complexes of linezolid (LINZ-1, LINZ-2 and LINZ-3) and three complexes of moxifloxacin (MOX-1, MOX-2 and MOX-3) with AC were designed and optimized, shown in Fig. S6. All the complexes, i.e., LINZ-1, LINZ-2, LINZ-3, MOX-1, MOX-3 show adsorption energies values greater in negative than -11 kcal mol^{-1} (Table S3) demonstrating shared interaction. The bond length values (1.75 to 2.23 Å and 1.73 to 2.54 Å) for linezolid and moxifloxacin complexes respectively with AC are quite stable. The strongest interactions occur when O atom of the C═O interact with the electropositive H atom of O─H in LINZ-1 having stable geometry, shortest bond length and lowest E_{ads} (-19.12 kcal mol^{-1}). Similarly, the MOX-3 is the most stable geometry among all the complexes of moxifloxacin with AC in which the O atom of the C═O interact with the electropositive H atom of O─H bond small bond length (1.73 and 2.54 Å) and lowest E_{ads} (-17.52 kcal mol^{-1}).

Frontier molecular orbital (FMO) and density of states (DOS) analyses[4] are used to investigate the strength of interactions between sorbate and sorbent. The energies of highest occupied molecular orbitals (HOMO) and lowest unoccupied molecular orbitals (LUMO) were simulated to compute the band gap (E_g). The results show that the band gap of the adsorbent (AC) decreases on interaction with adsorbates (MOX and LINZ). The band gap of AC is 2.45 eV and decreases for all complexes confirming the interaction between antibiotics and adsorbent. The density of states (DOS) analysis depicts the HOMO and LUMO states shown in Fig. S7 and the E_{HOMO} and E_{LUMO} values are listed in Table S3**,** reveal the appearance of new states i.e. HOMO and LUMO in the complexes which is responsible for the decrease in band gap values of the optimized complexes.

The FMO and DOS analyses suggests the presence of strong interaction between AC and MOX and LINZ validating the findings of E_{ads}.

The dipole moment helps in finding the presence of interaction between the adsorbate and adsorbent and increases for the complexes revealing the interaction of the adsorbent with adsorbates. The dipole moment of the complexes are listed in Table S3, depicts the increase for all the complexes of AC with linezolid and moxifloxacin compared to the dipole moment of virgin AC (5.91 Debye). The values of dipole moment justify the same order of the strength of interaction as shown by E_{ads} and bond length values. The E_{ads} values were higher for LINZ-1 and MOX-3 among the optimized configurations which is repeated by the greater increase of dipole moment for LINZ-1 and MOX-3 about 11.44 and 19.72 Debye respectively representing strong interaction in these two complexes among the optimized geometries.

Atoms in Molecules (AIM) analysis[5] has helped in understanding the nature and strength of interaction between activated carbon with linezolid and moxifloxacin. Topological parameters such as total electron energy densities (H_b), Laplacian ($\nabla^2\rho_b$) and density of all electrons (ρb) were calculated at bond critical points (BCPs) to study the behavior and strength of interaction of AC with linezolid and moxifloxacin. The bond critical point indicates interaction of AC with linezolid and moxifloxacin, shown in Fig. S8 and the values are given in Table S2. The numerical values of density of electrons (ρb) indicate the strength of interactions, greater the value of ρb for any complexes greater will be the strength of interaction and vice-versa. Among the various complexes of AC with MOX and LINZ, LINZ-1 and MOX-3 with highest value demonstrate the strong interaction in these complexes. The values of energy density (H_b) are positive for all complexes indicating the existence of shared interactions between adsorbent and adsorbate. Similarly, the positive values Laplacian energy ($\nabla^2\rho_b$) demonstrates the presence of electrostatic interaction at all

BCPs in the complexes of AC with linezolid and moxifloxacin. The presence of bond critical point between adsorbent and adsorbate evince the existence of strong interactions exists between AC and linezolid and moxifloxacin.

The interaction between activated carbon and drugs was studied in an ideal gaseous condition, so polarizable continuum model (PCM)[6] is applied to study the interaction in aqueous medium and the results are given in Table S3. Comparing the adsorption energy evolved in the gaseous phase and water phase, decrease in the negative values suggests the water influence on adsorption of antibiotics onto AC. Among various complexes (LINZ-1 and MOX-3) have the lowest E_{ads} in gas phase and water phase indicates the most stable complexes and strong interactive centers are found here. These findings are in agreement with band gap, AIM, DOS, FMO analyses and bond length demonstrate that hydrogen bonding and noncovalent interactions are involved in the adsorption MOX and LINZ onto AC.

Table S2 Bond Critical Points and Topological Parameters of Moxifloxacin and Linezolid Adsorbed onto Activated Carbon

System	BCP	Electron density (ρb)	laplacian ($\nabla^2\rho_b$)	Energy density (H_b)
LINZ-1	84	0.0380	0.1176	0.0005
LINZ-2	124	0.0223	0.0705	0.0003
	159	0.0142	0.0526	0.0012
LINZ-3	112	0.0291	0.1078	0.0003
	150	0.0057	0.0215	0.0009
MOX-1	143	0.0254	0.0913	0.0018
	144	0.0088	0.0319	0.0009
MOX-2	149	0.0175	0.0686	0.0001
MOX-3	141	0.0361	0.1267	0.0020
	142	0.0079	0.0269	0.0007

System	E_{ads} (Gas phase) (Kcal mol^{-1})	E_{ads} (Water phase) (Kcal mol^{-1})	Dipole moment (Debye)	E_{LUMO} (eV)	E_{HOMO} (eV)	E_g (eV)
KOH@AC	--------	--------	5.91	-2.64	-5.10	2.45
LINZ-1	-19.12	-7.26	11.44	-2.28	-4.50	2.22
LINZ-2	-17.48	-3.20	13.41	-2.48	-4.67	2.19
LINZ-3	-12.95	-4.10	5.10	-2.63	-4.82	2.18
MOX-1	-15.10	-2.45	15.48	-2.50	-4.64	2.14
MOX-2	-7.10	-1.10	15.41	-2.46	-4.67	2.21
MOX-3	-17.52	-4.87	19.72	-2.26	-4.41	2.14

Table S3 Adsorption Energy and Associated Parameters of Moxifloxacin and Linezolid Adsorbed onto Activated Carbon

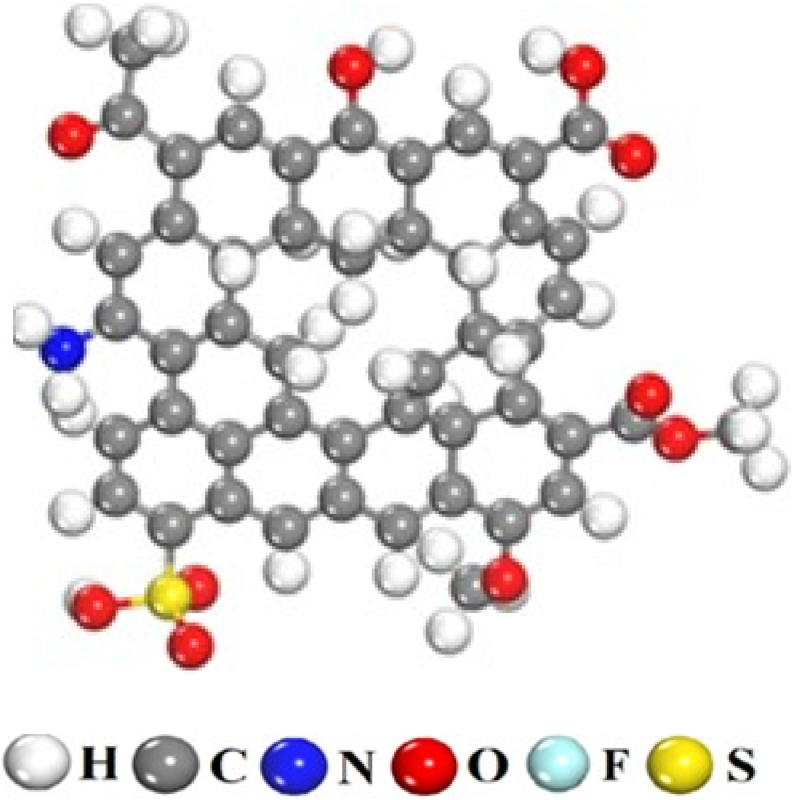

Figure S4 Optimized Structure of Activated Carbon

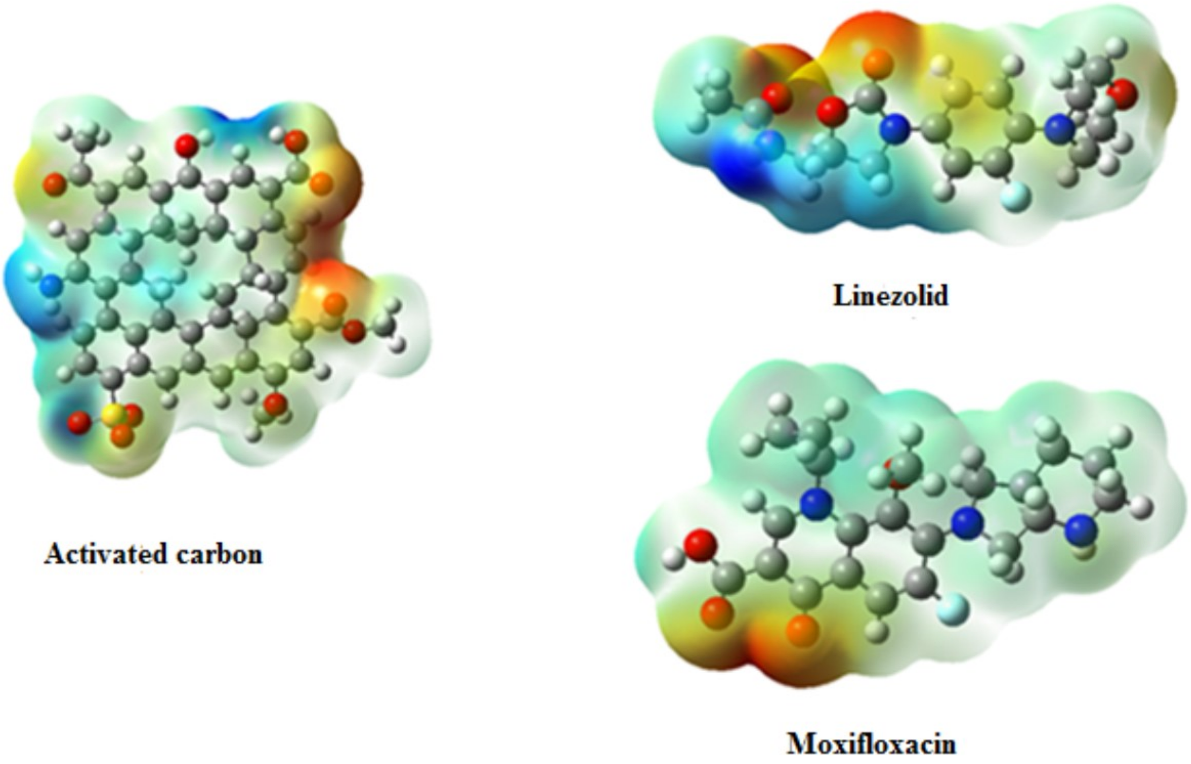

Figure S5 Molecular Electrostatic Potential Map of Activated Carbon, Moxifloxacin and Linezolid

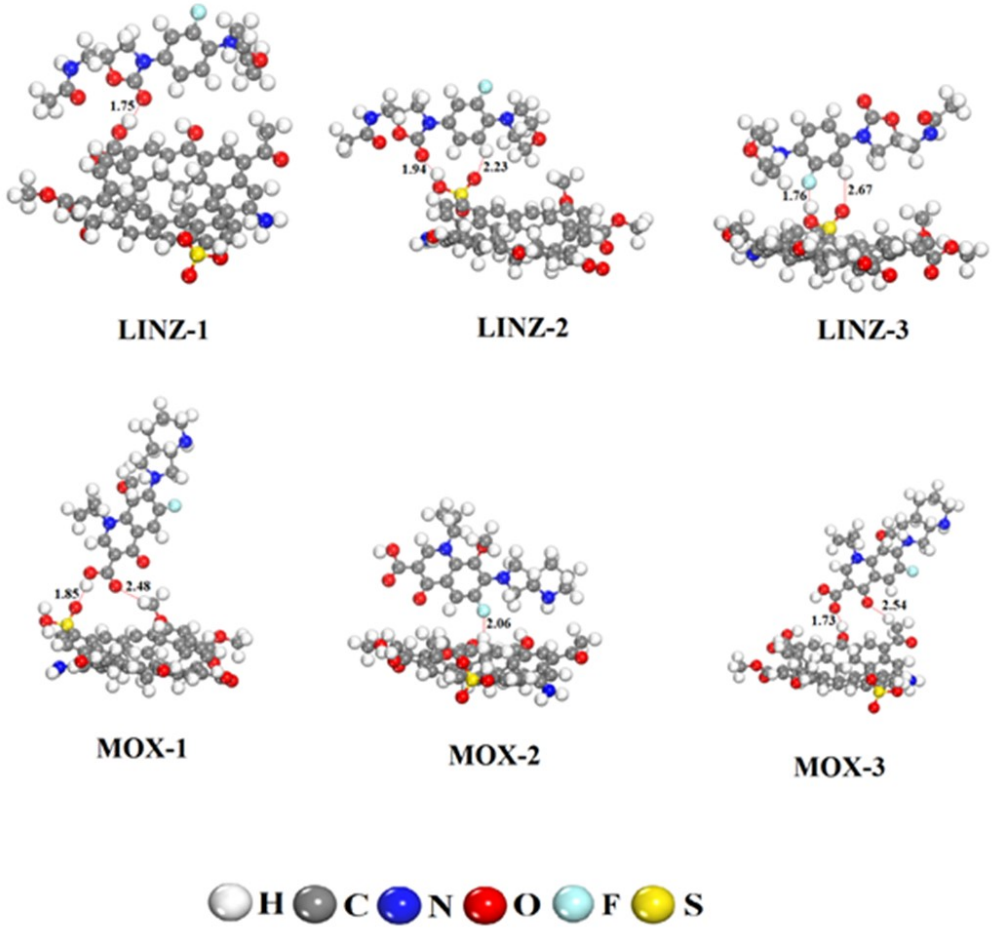

Figure S6 Optimized Geometries of Moxifloxacin and Linezolid Adsorbed onto Activated Carbon

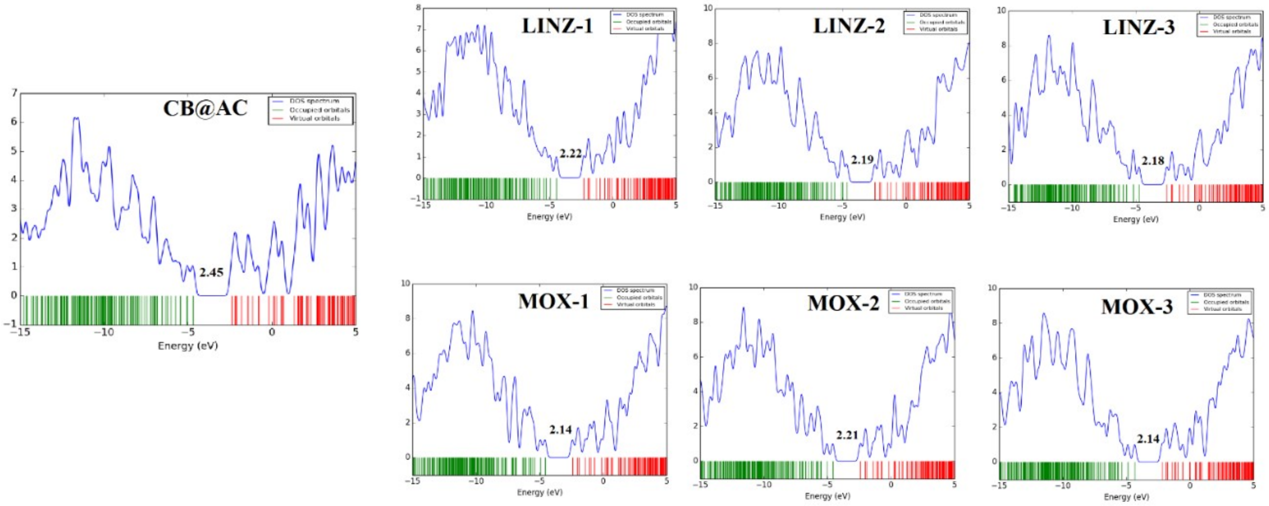

Figure S7 Density of States Analyses of Activated Carbon and Different Complexes of Moxifloxacin and Linezolid Adsorbed onto Activated Carbon

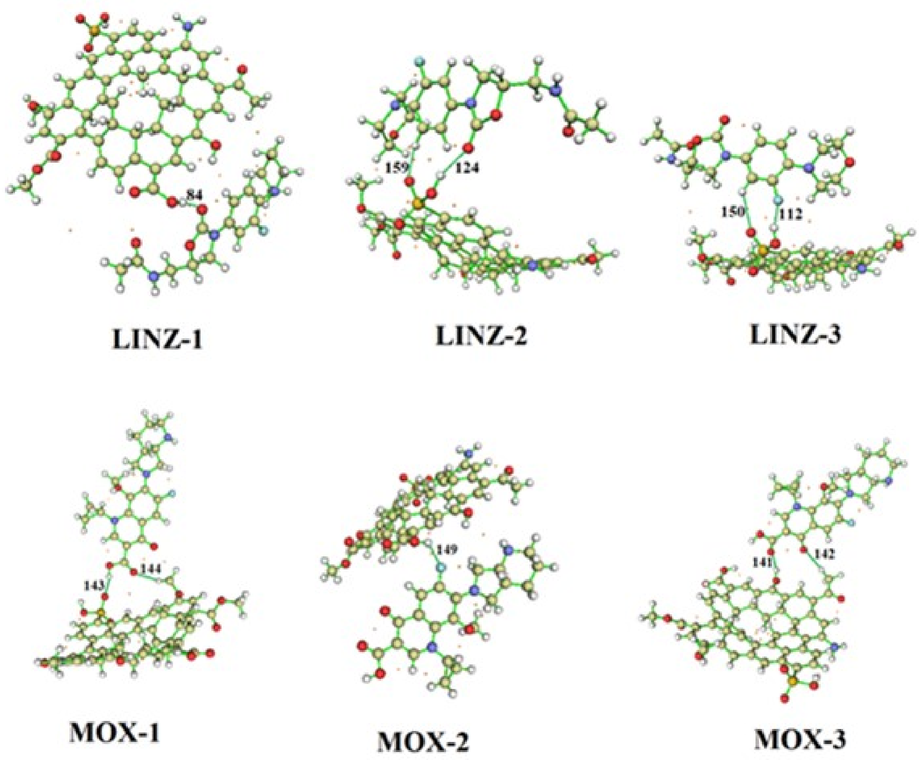

Figure S8 Atoms in Molecules Analyses of Moxifloxacin and Linezolid Adsorbed onto Activated Carbon

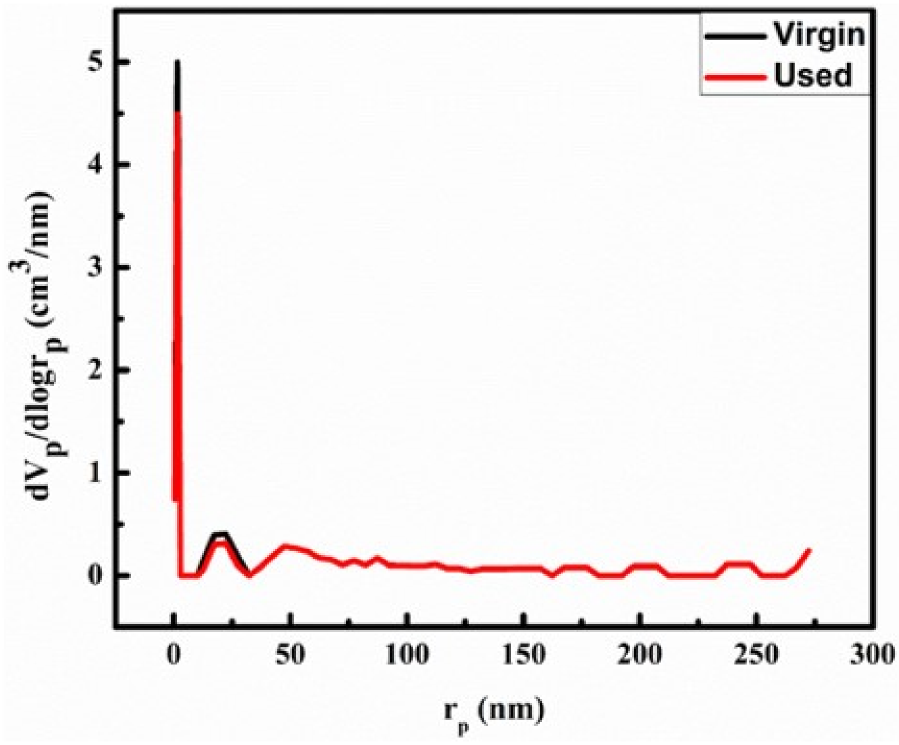

Fig. S9 Pore size distribution of the activated carbon before and after antibiotics adsorption

References

1 I. Ahmad, Adsorption of europium and thulium onto pine wood sawdust, University of Idaho, 2006.

2 A. A. Khan, I. Muhammad, R. Ahmad, I. Ahmad and N. Ullah, Quinone functionalized highly porous polymer as cathode material for Mg ion batteries: A DFT study, *J. Power Sourc.,* 2023, **580**, 233358.

3 K. A. Wilkinson, P. Sherwood, M. F. Guest and K. J. Naidoo, Acceleration of the GAMESS-UK electronic structure package on graphical processing units, *J. Compute. Chem.,* 2011, **32**(10), 2313-2318.

4 R. Peköz and Ş. Erkoç, Density functional theory study on the structural properties and energetics of Zn_mTe_n microclusters, *Physica E: Low-dimensional Systems and Nanostructures*, 2008, **40**(9), 2921-2930.

5 M. Blanco, A. Martín Pendás and E. Francisco, Interacting quantum atoms: a correlated energy decomposition scheme based on the quantum theory of atoms in molecules, *J. Chem. Theory Compute.,* 2005, **1**(6), 1096-1109.

6 S. Miertuš, E. Scrocco and J. Tomasi, Electrostatic interaction of a solute with a continuum. A direct utilizaion of AB initio molecular potentials for the prevision of solvent effects, *Chem. Phys.* 1981, **55**(1), 117-129.
